# Notch signaling pathway: a new target for neuropathic pain therapy

**DOI:** 10.1186/s10194-023-01616-y

**Published:** 2023-07-15

**Authors:** Yan Zhang, Tingting Wang, Sanlan Wu, Li Tang, Jia Wang, Jinghan Yang, Shanglong Yao, Yan Zhang

**Affiliations:** 1 Department of Anesthesiology, Union Hospital, Tongji Medical College, Huazhong University of Science and Technology, 1277 Jiefang Avenue, Wuhan, 430022 China; 2 Institute of Anesthesia and Critical Care Medicine, Union Hospital, Tongji Medical College, Huazhong University of Science and Technology, Wuhan, 430022 China; 3 Key Laboratory of Anesthesiology and Resuscitation (Huazhong University of Science and Technology), Ministry of Education, Wuhan, China; 4 Department of Pharmacy, Union Hospital, Tongji Medical College, Huazhong University of Science and Technology, Wuhan, 430022 China; 5 Department of Pain, Union Hospital, Tongji Medical College, Huazhong University of Science and Technology, 1277 Jiefang Avenue, Wuhan, 430022 China; 6 The Key Laboratory of Biomedical Information Engineering of Ministry of Education, Institute of Health and Rehabilitation Science, Research Center for Brain-Inspired Intelligence, School of Life Science and Technology, Xi’an Jiaotong University, The Key Laboratory of Neuro-Informatics & Rehabilitation En-Gineering of Ministry of Civil Affairs, Xi’an, Shaanxi P. R. China; 7 Department of Cardiovascular Surgery, Union Hospital, Tongji Medical College, Huazhong University of Science and Technology, 1277 Jiefang Ave, Wuhan, 430022 Hubei China

**Keywords:** Notch signaling pathway, Neuropathic pain, Neuroglia, Synaptic transmission, Calcium inward flow

## Abstract

The Notch gene, a highly evolutionarily conserved gene, was discovered approximately 110 years ago and has been found to play a crucial role in the development of multicellular organisms. Notch receptors and their ligands are single-pass transmembrane proteins that typically require cellular interactions and proteolytic processing to facilitate signal transduction. Recently, mounting evidence has shown that aberrant activation of the Notch is correlated with neuropathic pain. The activation of the Notch signaling pathway can cause the activation of neuroglia and the release of pro-inflammatory factors, a key mechanism in the development of neuropathic pain. Moreover, the Notch signaling pathway may contribute to the persistence of neuropathic pain by enhancing synaptic transmission and calcium inward flow. This paper reviews the structure and activation of the Notch signaling pathway, as well as its potential mechanisms of action, to provide novel insights for future treatments of neuropathic pain.

## Introduction

Neuropathic pain is chronic pain caused by lesion or diseases of the somatosensory system. Neuropathic pain has various etiologies and complex pathogenesis, with an incidence of 6.9% to 10% [1–4]. It is regarded as one of the most significant health problems in modern society [5]. The common characteristics of neuropathic pain include hyperalgesia, abnormal pain, and spontaneous pain [2, 3, 6]. Examples of common neuropathic pain encountered in clinics are postherpetic neuralgia, trigeminal neuralgia, complex regional pain syndrome, and diabetic peripheral neuralgia [7]. Drug therapy is currently the main therapeutic approach for neuropathic pain [7]. Despite the availability of a variety of drugs, the therapeutic effect is limited and accompanied by side effects. Clinically, there is still a need for the development of safe and effective drugs, as the current therapeutic options are not satisfactory [8].

The duration of neuropathic pain is often longer than the time of injury and some cases persist throughout life [9]. Chronic neuropathic pain may trigger concomitant anxiety and depression, significantly impairing patients' quality of life and contributing to the overall disease burden [4, 10]. Emerging research has indicated that the activation of glial cells and related signaling pathways assume an integral role in the development and sustenance of neuropathic pain [6].

The Notch gene, originally discovered by Morgan and his colleagues in the mutant drosophila in 1917, was named after the "notch" observed on the edge of the wings of drosophila melanocytosis resulting from partial function loss of this gene [11–13]. Notch homologs were subsequently identified in several metazoans such as Caenorhabditis elegans and Xenopus, all of which exhibited similar structures and signal components [14–16]. The Notch is highly conserved in evolution, widely found in both vertebrates and invertebrates, and plays a critical role in various physiological and pathological developmental processes including cell proliferation and migration, immune responses, angiogenesis, metastasis, memory, and neurological disorders, among others [17–20]. Therefore, the abnormality in the Notch signaling pathway can lead to serious pathological damage.

In recent decades, research has uncovered that activation of the Notch signaling pathway governs synaptic differentiation and transmission in the hippocampus [21, 22]. The Notch pathway is crucial in inducing and preserving neuropathic pain at the spinal level [22, 23]. The existing experimental evidence indicates that the activation of the Notch signaling pathway is involved in the pathological process of neuropathic pain. In this review, we present a systematic and comprehensive exposition of the structure, distribution, function, activation, and possible mechanisms for neuropathic pain of the Notch signaling pathway.

## Notch signaling pathway

### Notch signaling pathway structure

The Notch signaling pathway participates in numerous aspects of physical development, such as cell differentiation, tissue development, and organogenesis, as well as the occurrence and development of various diseases [17, 24]. Therefore, comprehending the structure of the Notch signaling pathway is an essential requirement for exploring the pathogenesis of these diseases. The Notch gene encodes a membrane protein receptor that is composed of three components: the Notch receptor, the Notch ligand (DSL protein), and the DNA binding sequence CSL (CBF1/Su(H)/Lag-1) [25, 26].

#### Notch receptor

The Notch receptor is a type I transmembrane protein with a single-pass domain, expressing on the cell membrane surface [27–30]. In mammals, there are four different Notch receptors, Notch1-4, each of which is encoded by a distinct gene. Notch1 and Notch2 are involved in the entire physical development and are widely expressed in many tissues of adult mammals, while Notch3 is predominantly expressed in vascular smooth muscle and pericytes, and Notch4 is highly expressed in endothelial cells [30]. It has been discovered that all Notch receptors, except Notch4, play a role in the development and maintenance of neuropathic pain. The expression of Notch1-3 in specific regions of the pain circuit is shown in Table 1. Notch1 is involved in regulating synaptic activity [31], Notch2 induces various intracellular responses associated with neuropathic pain [32], and Notch3 is associated with the differentiation and maturation of spinal cord neurons [33], the precise mechanisms require further investigation. All Notch receptors consist of three regions: the extracellular region (NEC), the transmembrane region (TM), and the intracellular region (NICD/ICN) [19, 26, 34, 35].

**Table 1:** Expression of Notch receptor in specific region in the pain circuit

Receptor	Region	Reference
Notch1	Dorsal root ganglia (DRG)	[31, 36–38]
	Spinal cord dorsal horn	[23, 39]
	Sciatica nerve	[39]
	Anterior cingulate cortex (ACC)	[40]
Notch2	Dorsal root ganglia (DRG)	[32, 33]
	Spinal cord dorsal horn	[32, 33]
Notch3	Spinal cord neuron precursors and/or immature neurons	[33, 41]

##### Extracellular region (NEC)

The extracellular region of Notch receptors is a structural domain comprised of 29–36 tandems epidermal growth factor (EGF) sequences and three cysteine-rich Lin Notch repeats [17, 20, 26, 29]. Its primary function is to initiate notch signaling by binding ligands. In mammals, Notch1 and Notch2 contain 36 EGF-like repeats; Notch3 contains 34 EGF-like repeats, and Notch4 contains 29 EGF-like repeats [42].

##### Transmembrane region (TM)

In the transmembrane region, an S3 cleavage is situated between glycine 1–1743 and valine 1–1744. The hydrolysis of the Notch receptor at the S3 site, including that of the Presenilin (mutant progerin) protein, cleaves the Notch receptor into the intracellular region ICN and a short transmembrane fragment. The Notch receptor's single transmembrane structural domain concludes with a C-terminal "stop translocation" signal comprising of 3–4 arginine/lysine (Arg/Lys) residues [43].

##### Intracellular domain (NICD/ICN)

The intracellular domain (NICD) of the Notch receptor localizes to the nucleus and represents the final outcome of Notch receptor activation [43]. This region primarily consists of one RAM (RBP2J kappa associated molecular) domain, seven anchor protein repeats (ankyrin repeats, ANK), two nuclear localization signals (NLS), one translation initiation region (translational active domain, TAD), and a PEST region (Proline, P (proline); Glutamate, E (glutamate); Serine, S (serine); Threonine, T (threonine)) [24, 29, 44]. The RAM region binds to DNA-binding protein (C2 promoter-binding factor (CBF)); while the ANK domains enhance Notch activation and facilitate interactions with other proteins. The PEST region plays an important role in the degradation of the Notch receptor [43, 44]. All four Notch receptors contain seven ANK structural domains and a PEST region. Notch1 and Notch2 possess the transcriptional activation domain (TAD), which is absent in Notch3 and Notch4. The NICD domain serves as the active form of the Notch receptor, and it’s binding to transcriptional activators initiates the activation of Notch target genes [19].

#### Notch ligand

Notch ligands, also known as DSL proteins, have been shown to exist in mammals in the form of five Notch ligands [43, 45, 46]: delta-like ligand 1 (DLL1), delta-like ligand 3 (DLL3), delta-like ligand 4 (DLL4), jagged-1 (JAG1) and jagged-2 (JAG2), each with both unique and redundant functions. The Delta-like family is distinguished from the Serrate family by the presence or absence of a cysteine-rich (CR) structural domain [47]. Notch ligands are transmembrane proteins that possess a conserved molecular structure, abbreviated as Delta/Serrate/Lag2, comprising an extracellular region with multiple EGF-R structural domains and DSL structural domains (cysteine-rich) that contain Notch receptor binding sites, thereby explaining Notch interactions, and short but distinctive intracellular structural domains [26, 30].

Notch ligands bind to Notch receptors on neighboring cells as well as on the same cell, which leads to activation or inhibition of Notch signaling [27, 48, 49]. This interaction occurs between the extracellular structural domains of Notch receptors and the DSL domains of Notch ligands.

DSL contains a 45 amino acid sequence consisting of six cysteines and three glycines [19]. In addition to the canonical DSL ligands, there are also atypical ligands that lack the DSL structural domain. These non-canonical ligands are a structurally diverse group of proteins that include integrally and glycosylphosphatidylinositol (GPI)-linked membrane proteins, which modulate Notch receptor activity [50].

#### DNA binding sequence CSL

CBF-1 (C-promoter binding factor-1) is a transcriptional repressor, called RBP-JK (recombination signal binding protein-Jk) in mammals [51], which recognizes and binds to a specific DNA sequence located at the promoter of Notch-inducible genes (GTGGGAA). It plays a key role in the Notch signaling pathway. Moreover, CBF-1 activates transcription by binding to the RAM and ANK structural domains of the Intracellular Domain of Notch (ICN), the binding of ICN displaces the SMRT co-inhibitor and the HDACase bound to it, thus relieving transcriptional repression. In the absence of NICD (ICN), Su(H)/CBFI recruits the blocker protein SMRT and histone deacetylase (HDAC) to repress gene transcription [37, 47, 52].

### Notch signaling pathway activation

#### The canonical NOTCH signaling pathway

**Fig. 1:**
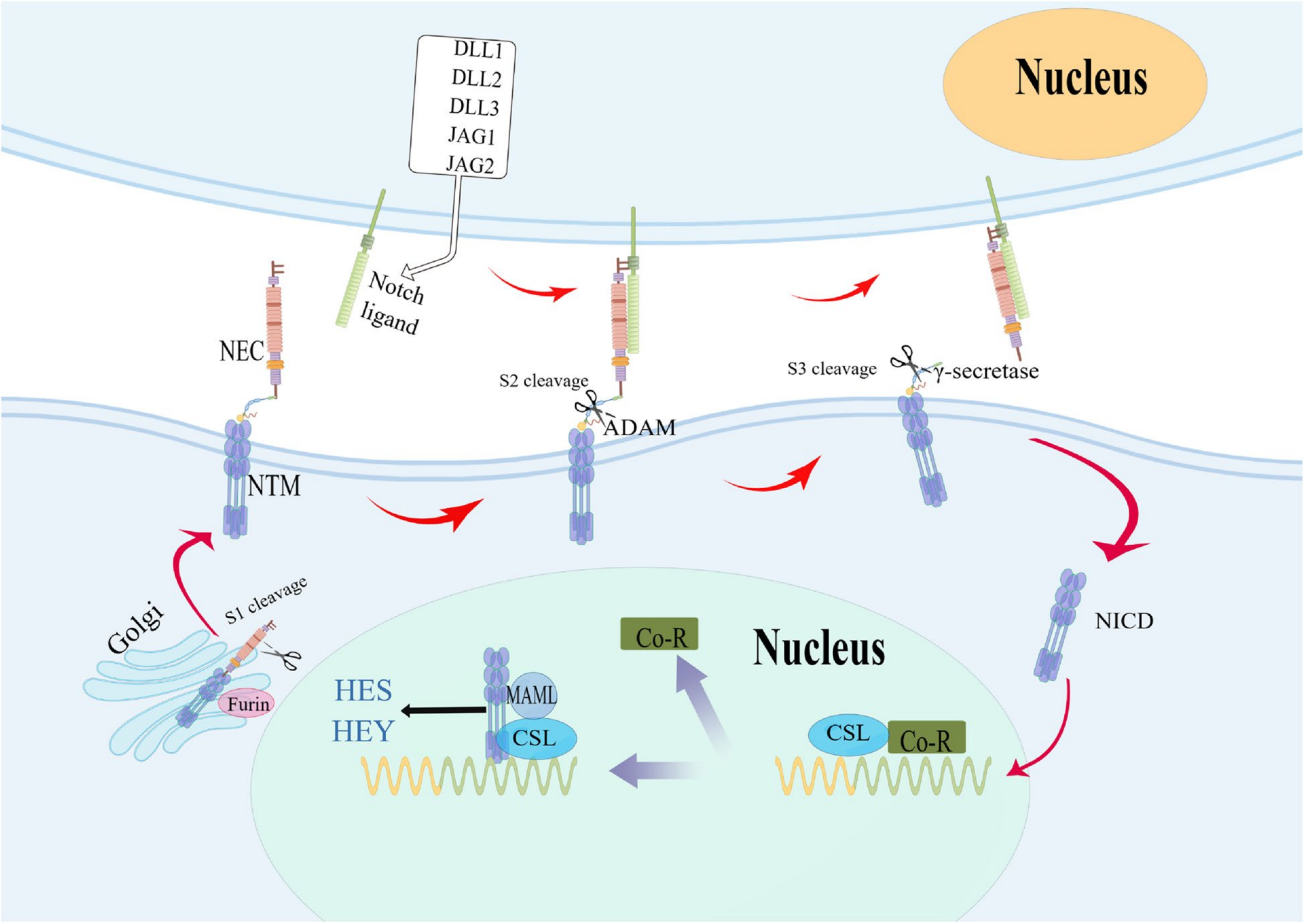
Schematic diagram of the canonical Notch signaling pathway. NEC, Notch extra-cellular domain; NTM, membrane-tethered intra-cellular domain; ADAM, A Disintegrin, and Metalloprotease; NICD, Notch intracellular domain; MAML, Mastermind; Co-R, Co-Repressors; CSL, CSL from CBF1 in vertebrates, suppressor of hairless in Drosophila, Lag-1 in Caenorhabditis elegans, RBP-Jκ in mammals

The canonical Notch signaling pathway is also known as the CBF-1/RBP-Jκ-dependent pathway, as depicted in Fig. 1. The activation of the Notch signaling pathway involves three cleavage events [53–55]: the first cleavage site, S1, takes place in the extracellular region between the arginine residue at 1654 and the tyramine residue in 1655. By the action of Furin protease in the Golgi complex, the Notch monomer is cleaved into two subunits: the Notch extracellular domain (NEC) and the Notch transmembrane fragment (NTM), which associate non-covalently through calcium-dependent bonds to form a heterodimeric Notch receptor complex located on the cell membrane surface. The second cleavage site, S2, is located in the extracellular proximal membrane region between residues 1710 alanine-1711 valine. When the Notch receptor binds to the ligand, it is cleaved into two fragments by the action of Metal Loprotease (ML)/Tumour Necrosis Factor-α converting enzyme (TACE) or Kuz, which belongs to ADAM (A Disintegrin and Metalloprotease) metalloproteinase family, to release the extracellular fragment. The N-terminal fragment (extracellular region) is phagocytosed by ligand-expressing cells, while the C-terminal cleavage product is further cleaved at the third cleavage site (S3) in the transmembrane region (located between residues 1743 glycine and 1744 valine) by γ-secretase, presenilin, and various cofactors, to release the activated form of Notch protein, NICD (ICN).

Upon NICD (ICN) entering the nucleus and binding CSL proteins (CBF1, Su(H), LAG1) through the RAM domain and CDC/ankyrin repeats, it recruits the nuclear transcriptional activator protein family MAML (mastermind-like) to form a ternary complex transcriptional activator (NICD-CSL-MAML). Once this complex is formed, Notch target genes encoding basic helix-loop-helix (bHLH) transcription factors such as HES (hairy/enhancer of split) and HEY (Hey-hairy/enhancer–of–split related with YRPW motif family members) are activated. These transcription factors promote the expression of downstream genes, thereby promoting cell proliferation and inhibiting cell differentiation [52, 56]. MAML (mastermind-like family members) acetylates histones by recruiting histone acetyltransferase 300p (HDAC) [53]. The binding of NICD to CSL proteins transforms CSL proteins from transcriptional repressors to transcriptional activators, thereby activating the transcription of the target gene.

#### The noncanonical NOTCH signaling pathway

The noncanonical Notch signaling pathway also known as the CSL non-dependent pathway involves interactions with other signaling pathways that occur upstream of the interactions between Notch ICD and CSL. The mature Notch receptor located on the cell membrane is activated partly by binding to its ligand and partly by endocytosed into the cytosol independent of the ligand. It then returns to the cell membrane to be degraded in the lysosome or activated in the endosome [57, 58]. Endosomes are known to contain ADAM and γ-secretase [47, 59]. Activation of the atypical Notch signaling pathway can be accomplished by binding non-typical ligands and does not require the excision of Notch receptors [44]. The ANK region of the Notch receptor binds to the intracellular zinc finger protein Deltex and represses the transcription factor E47.

### Notch signaling pathway regulation

#### At the extracellular level

The regulation of the notch signaling pathway at the extracellular level occurs in two distinct ways: firstly, through interactions with the extracellular segment of Notch, thereby impacting the binding of the normal Notch receptor to the ligand and subsequent signal transduction, involving factors such as Fringe, Wingless, Scabrous, among others. Secondly, active fragments of receptors and ligands are produced via the action of metalloproteinases, which disrupt the binding of normal Notch receptors and ligands, such as Kuzbanian, Fhrin, and similar proteins.

#### At the intracellular level

At the intracellular level, regulatory molecules are primarily regulated by two ways: proteolysis and protein–protein interactions. The primary regulatory molecules encompass Presenilins protease; Deltex, a protein containing a zinc finger that acts as a negative regulator of the Notch signaling pathway; and Numb, a membrane-bound protein.

#### At the nucleus level

At the intranuclear level, the expression of genes resulting from Notch activation is mainly regulated by two intranuclear proteins, Mastermind and Groucho. Mastermind, which has been found to bind to specific structures in chromatin, can upregulate or downregulate gene expression. On the other hand, Groucho, a non-basic helix-loop-helix (bHLH) protein, interacts with the DNA binding protein bHLH, E(sp1)/HES, to synergistically repress transcription. Studies have shown that Groucho is capable of binding to histone H3 on chromatin, causing transcriptional arrest.

## Notch mediates the mechanism of action of neuropathic pain

**Fig. 2:**
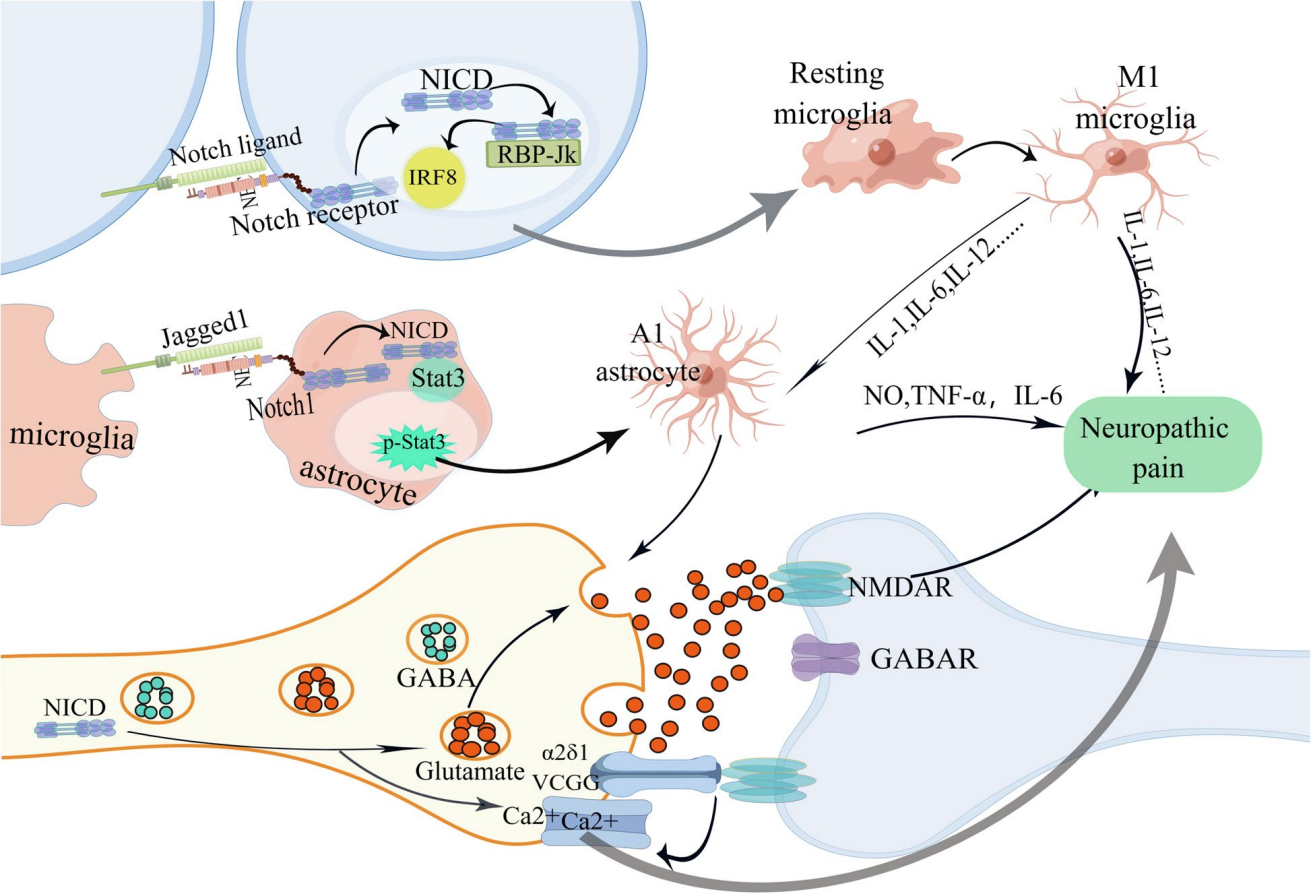
Mechanisms of Notch signaling pathways involved in neuropathic pain

According to the available literature, the Notch signaling pathway may play a role in the induction and maintenance of neuropathic pain through three mechanisms: activation of glial cells, enhancement of synaptic transmission, and alteration of ion channels (Fig. 2).

### Activated neuroglia

Neuroglia can be classified into two primary categories: microglia and macroglia (consisting of astrocytes and oligodendrocytes) [60]. The involvement of glial cells in the development and maintenance of neuropathic pain was initially reported in the 1990s [61–64]. Following nerve injury, astrocyte activation increases [65], releasing a plethora of factors such as nitric oxide (NO) [66], prostaglandins (PGs) [67], excitatory amino acids [68], cytokines (like TNF-α and IL-1β) [69] and ATP [70], which mediate pain hypersensitivity. Microglia is the primary immune cell in the central nervous system (CNS) and is the first and most critical line of immune-mediated defense. In neuropathic pain cases, microglia at spinal cord level become activated initially [71] and remain active for several weeks [72–74]. Activated microglia continuously release many pro-inflammatory mediators (like IL-1, IL-6, IL12), and express characteristic markers (such as CD11b, TLR4, CD14, and complement receptor 3 associated with the immune response (CR3)), resulting in a persistent pain state in the organism.

The Notch-RBP-Jκ signaling pathway has been discovered to regulate microglia polarization, neuroinflammation, and neuropathic pain by stimulating the translation of IRF8 [75–77]. Gui et al. [78] found that inhibition of the Notch-RBP-Jκ signaling pathway by Koumine reduced M1 polarization and inflammation in spinal microglia and improved diabetic neuropathic pain in rats. Following nerve injury, the Notch receptor combines with its ligand, which activates the Notch signaling pathway, and released an active signal fragment NICD [79, 80]. The NICD then enters the nucleus and forms a transcriptional activation complex with the transcription factor RBP-Jκ, which stimulates translation and enhances the synthesis of IRF8. This, in turn, contributes to the production of pro-inflammatory cytokines, leading to rapid microglia polarization [76, 79, 81] and the induction of neuropathic pain. The Notch pathway interacts with many other signaling pathways and studies have shown that transcription activator 3 (Stat3) is an important transcription factor in A1 astrocytes [82]. Li et al. [83] found that Jagged1, the ligand of the Notch receptor, is expressed with microglia and neurons, while the Notch1 receptor is expressed on astrocytes and neurons. Upon nerve injury, microglia are activated and Jagged1 located in microglia binds to Notch receptors on astrocytes, inducing Notch pathway activation. Qian et al. [84] found that upon activation of the Notch pathway, the activating fragment NICD binds to Stat3 to promote its phosphorylation and then enters the nucleus to promote the transformation of A1 (pro-inflammatory) astrocytes, which plays a significant role in the maintenance of neuropathic pain.

### Altering ion channels

The voltage-gated calcium channel (VGCC) plays a significant role in the transduction of neuropathic pain. The accessory α2δ1 subunit of VGCC is predominantly present in the presynaptic terminal and is associated with synaptic development and the progression of neuropathic pain through its interaction with TSP secreted by astrocytes [85, 86]. Moreover, the α2δ1 subunit interacts directly with N-methyl-D-aspartate (NMDA) receptors to initiate presynaptic NMDA receptor activation [87], which is integral to neurotransmitter release, synaptic plasticity and neuropathic pain [88, 89]. Following nerve injury, upregulation of α2δ subunit expression occurs [90], and α2δ calcium channels are activated by conjugation with TSP or NMDA receptors at the presynaptic end of neurons, triggering an influx of Ca2 + and increased neuronal excitability. This, in turn, stimulates downstream signaling of protein kinase C (PKC) and transient receptor potential ankyrin 1 (TRPA1) and transient receptor potential vanilloid 1 (TRPV1) channel, ultimately inducing neuropathic pain.

Several studies have shown that the Notch signalling pathway enhances calcium influx in dorsal root ganglia (DRG) [91]. Upon nerve injury, activation of the Notch signaling pathway involves γ-secretase in the processing of Notch receptor [92], the γ-secretase activity requires progerin, and its mutants can impede proteolytic processing of Notch receptors, resulting in changes in store-operated Ca2 + entry (SOCE) [93, 94]. SOCE plays a role in Ca2 + endocytosis [95, 96], sustains Ca2 + elevation after store mobilization, and enhances secretion in certain cell types [97]. SOCE also activates transcription [98], alters synaptic transmission [99], and enhances Ca2 + endocytosis [100], leading to heightened neuronal excitability, which then triggers a subsequent cascade of signaling events that induce neuropathic pain.

### Enhancing synaptic transmission

Sustained stimulation due to nerve injury and inflammation can result in enhanced synaptic transmission, diminished inhibition, and synaptic plasticity [101], along with activation of Aδ and C fibers releasing the excitatory neurotransmitter, glutamate. Glutamate combines with N-methyl-D-aspartate (NMDA) receptors, and the decreased activation threshold of NMDA receptors results in altered excitability of sensory neurons in the dorsal horn of the spinal cord, giving rise to a persistent rise in the frequency of synaptic activity. This is evidenced by increased spontaneous and evoked neuronal firing, expanded sensory fields, and ultimately, the development of spontaneous pain and nociceptive hyperalgesia [102].

Studies have shown that Notch1 affects the expression and composition of NMDA receptors and that increased Notch1 expression leads to increased glutamatergic transmission [21]. Research indicated that Notch1 is located in the synapse [22], is upregulated in response to neuronal activity, and amplifies neuronal excitation and synaptic transmission [103–105]. This amplification results in an imbalance of glutamate/GABA transmission, leading to central sensitization and persistent pain after nerve injury.

## Discussion

Neuropathic pain is a chronic pain triggered by various factors including incisions, autoimmune diseases, nerve compression, and channel lesions [106], with complex pathological mechanisms. In the past decades, there has been a great deal of clinical and basic medical research into neuropathic pain. However, most of the potential pathological mechanisms are not yet accurately understood owing to the complex and diverse etiology. The current clinical treatment for neuropathic pain mainly involves analgesic drugs, surgery, spinal cord stimulation [107], transcutaneous electrical nerve stimulation [108], and other technical means, with a lack of specific treatment targeting the underlying mechanism.

Recently, studies have identified the Notch signaling pathway as a new target in neuropathic pain pathogenesis [69, 83, 109], Notch signaling is activated during the development of neuropathic pain, activating astrocytes and microglia and causing mechanical allodynia. It is well known that mechanical allodynia is a major feature of neuropathic pain. Li et al. [83], and Xie et al. [75], found that dose-dependent administration of Jagged1 (a ligand for the Notch receptor) resulted in significant activation of both Notch signaling and glial cells, inducing mechanical allodynia. Also, Duan et al. [40] discovered that the downregulation of Hes1, an effector of Notch signaling, attenuated neuropathic pain. Moreover, Sun et al. [23], Yang et al. [81], and Qin et al. [39] found that DAPT (an inhibitor of Notch signaling) down-regulated Notch expression, inhibited glial cell transformation and reversed mechanical allodynia. This suggests that JAG-1, Hes-1 and γ-secretase could be targets of the Notch signaling pathway. However, only DAPT, an inhibitor targeting γ-secretase, has been studied, and no inhibitors targeting JAG-1 and Hes-1 have been reported in the literature.

Current studies have shown that DAPT enters the rat or mouse mainly by both intrathecal catheter and intraperitoneal injection, targeting the gamma-secretase, a key enzyme in the Notch signaling pathway. Studies have shown that both modes of administration have been studied experimentally in spinal cord tissue or in the DRG or ACC. Whether Notch inhibitors that do not cross the blood–brain barrier can reduce pain has not been reported in the literature. More importantly, the results of spinal transcriptome sequencing in our previous experimental study showed that Notch signaling pathway was significantly upregulated and Notch1 gene expression was significantly increased in the SNI model, once again confirming that the Notch signaling pathway plays a key role in neuropathic pain.

Thus, the discovery of the Notch signaling pathway as a novel aspect of neuropathic pain pathogenesis and its potential targeting holds promise. We have compiled a list of potential FDA-approved therapeutic agents targeting Notch for other conditions that may be used for neuropathic pain treatment (Table 2).

**Table 2:** Potential therapeutic agents against Notch

Therapeutics	Disease	Target	Reference
Venetoclax	breast cancer	γ-secretase	[110]
Ciclopirox (CPX)	Bladder cancer		[111]
Cucurbitacin B and I	Colorectal cancer (CRC)		[112]
RO4929097	Advanced Sarcoma		[113]
BMS-906024	Lung cancer		[114]
dibenzazepine (DBZ)	Obesity		[115]
Crenigacestat (LY3039478)	Calcific aortic valve disease (CAVD)		[116]
	T-cell acute lymphoblastic leukemia (T-ALL)/T-cell lymphoblastic lymphoma (T-LBL)		[117]

## Future perspective

In this review we systematically describe the mechanisms of Notch signaling pathways in the induction and maintenance of neuropathic pain, intimately related to excitatory synaptic transmission, neuroglia activation, and calcium inward flow, and there may be undiscovered pathogenic mechanisms. Recently, Numerous studies on the mechanisms of the Notch signaling pathway in the development and maintenance of neuropathic pain have revealed possible therapeutic targets. At present, most studies have been conducted at the animal level. Moreover, drug treatments targeting the Notch signaling pathway have only been experimented with in other disease models. Therefore, further future studies are necessary to be conducted both clinically and at a basic level to provide data to support the targeted treatment of neuropathic pain.

## Data Availability

Not applicable.

## References

[1] St John Smith  E (2018). Advances in understanding nociception and neuropathic pain. J Neurol.

[2] Baron R (2017). Peripheral neuropathic pain: a mechanism-related organizing principle based on sensory profiles. Pain.

[3] Alles SRA, Smith PA (2018). Etiology and pharmacology of neuropathic pain. Pharmacol Rev.

[4] Cohen SP, Mao J (2014). Neuropathic pain: mechanisms and their clinical implications. BMJ.

[5] Maia RD (2017). Recent trends in neuropathic pain patents. Expert Opin Ther Pat.

[6] Inoue K, Tsuda M (2018). Microglia in neuropathic pain: cellular and molecular mechanisms and therapeutic potential. Nat Rev Neurosci.

[7] Jensen TS, Finnerup NB (2014). Allodynia and hyperalgesia in neuropathic pain: clinical manifestations and mechanisms. Lancet Neurol.

[8] Bouhassira D, Attal N (2018). Emerging therapies for neuropathic pain: new molecules or new indications for old treatments?. Pain.

[9] Stratton HJ, Khanna R (2020). Sculpting dendritic spines during initiation and maintenance of neuropathic pain. J Neurosci.

[10] Scholz J (2019). The IASP classification of chronic pain for ICD-11: chronic neuropathic pain. Pain.

[11] Metz CW, Bridges CB (1917). Incompatibility of mutant races in drosophila. Proc Natl Acad Sci U S A.

[12] Mohr OL (1919). Character changes caused by mutation of an entire region of a chromosome in drosophila. Genetics.

[13] Bridges CB (1916). Non-disjunction as proof of the chromosome theory of heredity (Concluded). Genetics.

[14] Austin J, Kimble J (1989). Transcript analysis of glp-1 and lin-12, homologous genes required for cell interactions during development of C. elegans. Cell.

[15] Coffman C, Harris W, Kintner C (1990). Xotch, the Xenopus homolog of Drosophila notch. Science.

[16] Stubbs JD, Lekutis C, Singer KL, Bui A, Yuzuki D, Srinivasan U, Parry G (1990). cDNA cloning of a mouse mammary epithelial cell surface protein reveals the existence of epidermal growth factor-like domains linked to factor VIII-like sequences. Proc Natl Acad Sci U S A.

[17] Zhou B (2022). Notch signaling pathway: architecture, disease, and therapeutics. Signal Transduct Target Ther.

[18] Golub R (2021). The Notch signaling pathway involvement in innate lymphoid cell biology. Biomed J.

[19] Gao Y (2023). The role of Notch signaling pathway in metabolic bone diseases. Biochem Pharmacol.

[20] Castro RC (2021). Notch signaling pathway in infectious diseases: role in the regulation of immune response. Inflamm Res.

[21] Brai E (2015). Notch1 regulates hippocampal plasticity through interaction with the Reelin pathway, glutamatergic transmission and CREB Signaling. Front Cell Neurosci.

[22] Alberi L (2011). Activity-induced Notch signaling in neurons requires Arc/Arg3.1 and is essential for synaptic plasticity in hippocampal networks. Neuron.

[23] Sun YY, Li L, Liu XH, Gu N, Dong HL, Xiong L (2012). The spinal notch signaling pathway plays a pivotal role in the development of neuropathic pain. Mol Brain.

[24] Hashemi M (2022). Non-coding RNAs targeting notch signaling pathway in cancer: From proliferation to cancer therapy resistance. Int J Biol Macromol.

[25] Luo Z (2019). Notch signaling in osteogenesis, osteoclastogenesis, and angiogenesis. Am J Pathol.

[26] Li L (2017). Notch signaling pathway networks in cancer metastasis: a new target for cancer therapy. Med Oncol.

[27] Ballhause TM (2021). Relevance of notch signaling for bone metabolism and regeneration. Int J Mol Sci.

[28] Aggarwal V (2021). NOTCH signaling: Journey of an evolutionarily conserved pathway in driving tumor progression and its modulation as a therapeutic target. Crit Rev Oncol Hematol.

[29] Shim YS, Lee HS, Hwang JS (2022). Aberrant notch signaling pathway as a potential mechanism of central precocious puberty. Int J Mol Sci.

[30] Weinmaster G, Kintner C (2003). Modulation of notch signaling during somitogenesis. Annu Rev Cell Dev Biol.

[31] Chen T (2017). Interactions of Notch1 and TLR4 signaling pathways in DRG neurons of in vivo and in vitro models of diabetic neuropathy. Sci Rep.

[32] Zhang Y (2021). Circ_0005075 targeting miR-151a-3p promotes neuropathic pain in CCI rats via inducing NOTCH2 expression. Gene.

[33] Rusanescu G, Mao J (2014). Notch3 is necessary for neuronal differentiation and maturation in the adult spinal cord. J Cell Mol Med.

[34] Vlachakis D (2020). An updated evolutionary study of the Notch family reveals a new ancient origin and novel invariable motifs as potential pharmacological targets. PeerJ.

[35] Chen W (2021). The Notch signaling pathway regulates macrophage polarization in liver diseases. Int Immunopharmacol.

[36] Wiszniak S, Schwarz Q (2019). Notch signalling defines dorsal root ganglia neuroglial fate choice during early neural crest cell migration. BMC Neurosci.

[37] Wang D, Lu J, Xu X, Yuan Y, Zhang Y, Xu J, Chen H, Liu J, Shen Y, Zhang H (2021). Satellite glial cells give rise to nociceptive sensory neurons. Stem Cell Rev Rep.

[38] McGraw HF, Snelson CD, Prendergast A, Suli A, Raible DW (2012). Postembryonic neuronal addition in zebrafish dorsal root ganglia is regulated by Notch signaling. Neural Dev.

[39] Qin B, Li Y, Liu X, Gong D, Zheng W (2020). Notch activation enhances microglial CX3CR1/P38 MAPK pathway in rats model of vincristine-induced peripheral neuropathy. Neurosci Lett.

[40] Duan H, Shen F, Li L, Tu Z, Chen P, Chen P, Wang Z, Liang W, Wang Y (2021). Activation of the Notch signaling pathway in the anterior cingulate cortex is involved in the pathological process of neuropathic pain. Pain.

[41] Rusanescu G (2016). Adult spinal cord neurogenesis: A regulator of nociception. Neurogenesis (Austin).

[42] Previs RA (2015). Molecular pathways: translational and therapeutic implications of the Notch signaling pathway in cancer. Clin Cancer Res.

[43] Kopan R, Ilagan MX (2009). The canonical Notch signaling pathway: unfolding the activation mechanism. Cell.

[44] Andersson ER, Sandberg R, Lendahl U (2011). Notch signaling: simplicity in design, versatility in function. Development.

[45] Yuan X (2015). Notch signaling: an emerging therapeutic target for cancer treatment. Cancer Lett.

[46] Louvi A, Artavanis-Tsakonas S (2006). Notch signalling in vertebrate neural development. Nat Rev Neurosci.

[47] Hori K, Sen A, Artavanis-Tsakonas S (2013). Notch signaling at a glance. J Cell Sci.

[48] D'Souza B, Meloty-Kapella L, Weinmaster G (2010). Canonical and non-canonical Notch ligands. Curr Top Dev Biol.

[49] Siebel C, Lendahl U (2017). Notch signaling in development, tissue homeostasis, and disease. Physiol Rev.

[50] D'Souza B, Miyamoto A, Weinmaster G (2008). The many facets of Notch ligands. Oncogene.

[51] BeLow M, Osipo C (2020). Notch signaling in breast cancer: a role in drug resistance. Cells.

[52] McIntyre B, Asahara T, Alev C (2020). Overview of basic mechanisms of notch signaling in development and disease. Adv Exp Med Biol.

[53] Wang H (2015). The role of Notch receptors in transcriptional regulation. J Cell Physiol.

[54] Vanderbeck A, Maillard I (2021). Notch signaling at the crossroads of innate and adaptive immunity. J Leukoc Biol.

[55] D'Assoro AB (2022). Roles of notch signaling in the tumor microenvironment. Int J Mol Sci.

[56] Masek J, Andersson ER (2017). The developmental biology of genetic Notch disorders. Development.

[57] Pamarthy S (2018). The curious case of vacuolar ATPase: regulation of signaling pathways. Mol Cancer.

[58] Conner SD (2016). Regulation of notch signaling through intracellular transport. Int Rev Cell Mol Biol.

[59] Steinbuck MP, Arakcheeva K, Winandy S (2018). Novel TCR-Mediated Mechanisms of Notch Activation and Signaling. J Immunol.

[60] Yang QH (2022). Non-invasive brain stimulation for central neuropathic pain. Front Mol Neurosci.

[61] Vicario N (2022). Mu and delta opioid receptor targeting reduces connexin 43-based heterocellular coupling during neuropathic pain. Int J Mol Sci.

[62] Sheu ML (2022). Modulation of aryl hydrocarbon receptor expression alleviated neuropathic pain in a chronic constriction nerve injury animal model. Int J Mol Sci.

[63] Colburn RW, DeLeo JA, Rickman AJ, Yeager MP, Kwon P, Hickey WF (1997). Dissociation of microglial activation and neuropathic pain behaviors following peripheral nerve injury in the rat. J Neuroimmunol.

[64] Colburn RW, Rickman AJ, DeLeo JA (1999). The effect of site and type of nerve injury on spinal glial activation and neuropathic pain behavior. Exp Neurol.

[65] Garrison CJ, Dougherty PM, Kajander KC, Carlton SM (1991). Staining of glial fibrillary acidic protein (GFAP) in lumbar spinal cord increases following a sciatic nerve constriction injury. Brain Res.

[66] Liu JS, John GR, Sikora A, Lee SC, Brosnan CF (2000). Modulation of interleukin-1beta and tumor necrosis factor alpha signaling by P2 purinergic receptors in human fetal astrocytes. J Neurosci.

[67] Ghilardi JR (2004). Constitutive spinal cyclooxygenase-2 participates in the initiation of tissue injury-induced hyperalgesia. J Neurosci.

[68] Duan S, Anderson CM, Keung EC, Chen Y, Chen Y, Swanson RA (2003). P2X7 receptor-mediated release of excitatory amino acids from astrocytes. J Neurosci.

[69] Milligan ED, Twining C, Chacur M, Biedenkapp J, O'Connor K, Poole S, Tracey K, Martin D, Maier SF, Watkins LR (2003). Spinal glia and proinflammatory cytokines mediate mirror-image neuropathic pain in rats. J Neurosci.

[70] Queiroz G, Gebicke-Haerter PJ, Schobert A, Starke K, von Kügelgen I (1997). Release of ATP from cultured rat astrocytes elicited by glutamate receptor activation. Neuroscience.

[71] Tanga FY, Raghavendra V, DeLeo JA (2004). Quantitative real-time RT-PCR assessment of spinal microglial and astrocytic activation markers in a rat model of neuropathic pain. Neurochem Int.

[72] Clark AK (2007). Role of spinal microglia in rat models of peripheral nerve injury and inflammation. Eur J Pain.

[73] Clark AK, Yip PK, Grist J, Gentry C, Staniland AA, Marchand F, Dehvari M, Wotherspoon G, Winter J, Ullah J, Bevan S, Malcangio M (2007). Inhibition of spinal microglial cathepsin S for the reversal of neuropathic pain. Proc Natl Acad Sci U S A..

[74] Coyle DE (1998). Partial peripheral nerve injury leads to activation of astroglia and microglia which parallels the development of allodynic behavior. Glia.

[75] Xie K (2015). Notch signaling activation is critical to the development of neuropathic pain. BMC Anesthesiol.

[76] Wu F (2018). Simvastatin alters M1/M2 polarization of murine BV2 microglia via Notch signaling. J Neuroimmunol.

[77] Cheng Z (2019). Inhibition of notch1 signaling alleviates endotoxin-induced inflammation through modulating retinal microglia polarization. Front Immunol.

[78] Jin GL, Hong LM, Liu HP, Yue RC, Shen ZC, Yang J, Xu Y, Huang HH, Li Y, Xiong BJ, Su YP, Yu CX (2021). Koumine modulates spinal microglial M1 polarization and the inflammatory response through the Notch-RBP-Jκ signaling pathway, ameliorating diabetic neuropathic pain in rats. Phytomedicine.

[79] Xu H (2012). Notch-RBP-J signaling regulates the transcription factor IRF8 to promote inflammatory macrophage polarization. Nat Immunol.

[80] Pierfelice T, Alberi L, Gaiano N (2011). Notch in the vertebrate nervous system: an old dog with new tricks. Neuron.

[81] Yang C (2017). Minocycline attenuates the development of diabetic neuropathy by inhibiting spinal cord Notch signaling in rat. Biomed Pharmacother.

[82] Reichenbach N, Delekate A, Plescher M, Schmitt F, Krauss S, Blank N, Halle A, Petzold GC (2019). Inhibition of Stat3-mediated astrogliosis ameliorates pathology in an Alzheimer's disease model. EMBO Mol Med.

[83] Li DY, Gao SJ, Sun J, Zhang LQ, Wu JY, Song FH, Liu DQ, Zhou YQ, Mei W (2022). Notch signaling activation contributes to paclitaxel-induced neuropathic pain via activation of A1 astrocytes. Eur J Pharmacol.

[84] Qian D, Li L, Rong Y, Liu W, Wang Q, Zhou Z, Gu C, Huang Y, Zhao X, Chen J, Fan J, Yin G (2019). Blocking Notch signal pathway suppresses the activation of neurotoxic A1 astrocytes after spinal cord injury. Cell Cycle.

[85] Wang J (2021). Cocaine triggers astrocyte-mediated synaptogenesis. Biol Psychiatry.

[86] Risher WC (2018). Thrombospondin receptor alpha2delta-1 promotes synaptogenesis and spinogenesis via postsynaptic Rac1. J Cell Biol.

[87] Chen J (2018). The alpha2delta-1-NMDA receptor complex is critically involved in neuropathic pain development and gabapentin therapeutic actions. Cell Rep.

[88] Huang Y (2020). Calcineurin inhibition causes α2δ-1–mediated tonic activation of synaptic NMDA receptors and pain hypersensitivity. J Neurosci.

[89] Deng M, Chen SR, Pan HL (2019). Presynaptic NMDA receptors control nociceptive transmission at the spinal cord level in neuropathic pain. Cell Mol Life Sci.

[90] Bauer CS, Rahman W, Tran-van-Minh A, Lujan R, Dickenson AH, Dolphin AC (2010). The anti-allodynic alpha(2)delta ligand pregabalin inhibits the trafficking of the calcium channel alpha(2)delta-1 subunit to presynaptic terminals in vivo. Biochem Soc Trans.

[91] Shideman CR, Reinardy JL, Thayer SA (2009). gamma-Secretase activity modulates store-operated Ca2+ entry into rat sensory neurons. Neurosci Lett.

[92] Fortini ME (2002). Gamma-secretase-mediated proteolysis in cell-surface-receptor signalling. Nat Rev Mol Cell Biol.

[93] Herms J (2003). Capacitive calcium entry is directly attenuated by mutant presenilin-1, independent of the expression of the amyloid precursor protein. J Biol Chem.

[94] Yoo AS, Cheng I, Chung S, Grenfell TZ, Lee H, Pack-Chung E, Handler M, Shen J, Xia W, Tesco G, Saunders AJ, Ding K, Frosch MP, Tanzi RE, Kim TW (2000). Presenilin-mediated modulation of capacitative calcium entry. Neuron.

[95] Putney JW (2003). Capacitative calcium entry in the nervous system. Cell Calcium.

[96] Bouron A (2000). Activation of a capacitative Ca(2+) entry pathway by store depletion in cultured hippocampal neurones. FEBS Lett.

[97] Fomina AF, Nowycky MC (1999). A current activated on depletion of intracellular Ca2+ stores can regulate exocytosis in adrenal chromaffin cells. J Neurosci.

[98] Loh C, Carew JA, Kim J, Hogan PG, Rao A (1996). T-cell receptor stimulation elicits an early phase of activation and a later phase of deactivation of the transcription factor NFAT1. Mol Cell Biol.

[99] Baba A, Yasui T, Fujisawa S, Yamada RX, Yamada MK, Nishiyama N, Matsuki N, Ikegaya Y (2003). Activity-evoked capacitative Ca2+ entry: implications in synaptic plasticity. J Neurosci.

[100] Fanger CM, Hoth M, Crabtree GR, Lewis RS (1995). Characterization of T cell mutants with defects in capacitative calcium entry: genetic evidence for the physiological roles of CRAC channels. J Cell Biol.

[101] Flor H (2014). Psychological pain interventions and neurophysiology: Implications for a mechanism-based approach. Am Psychol.

[102] Luo XQ (2014). Tyrosine phosphorylation of the NR2B subunit of the NMDA receptor in the spinal cord contributes to chronic visceral pain in rats. Brain Res.

[103] Narita M (2011). Sleep disturbances in a neuropathic pain-like condition in the mouse are associated with altered GABAergic transmission in the cingulate cortex. Pain.

[104] Chen ZY (2016). Attenuation of neuropathic pain by inhibiting electrical synapses in the anterior cingulate cortex. Anesthesiology.

[105] Chen T (2018). Top-down descending facilitation of spinal sensory excitatory transmission from the anterior cingulate cortex. Nat Commun.

[106] Bannister K (2020). Neuropathic Pain: Mechanism-Based Therapeutics. Annu Rev Pharmacol Toxicol.

[107] Sun L (2021). Spinal cord stimulation and treatment of peripheral or central neuropathic pain: mechanisms and clinical application. Neural Plast.

[108] Mokhtari T (2020). Transcutaneous electrical nerve stimulation in relieving neuropathic pain: basic mechanisms and clinical applications. Curr Pain Headache Rep.

[109] Li C, Huang S, Zhou W, Xie Z, Xie S, Li M (2022). Effects of the notch signaling pathway on secondary brain changes caused by spinal cord injury in mice. Neurochem Res.

[110] Pathak Y, Camps I, Mishra A, Tripathi V (2022) Targeting notch signaling pathway in breast cancer stem cells through drug repurposing approach. Mol Divers. 10.1007/s11030-022-10561-y 10.1007/s11030-022-10561-y 36376717

[111] Weir SJ, Dandawate P, Standing D, Bhattacharyya S, Ramamoorthy P, Rangarajan P, Wood R, Brinker AE, Woolbright BL, Tanol M, Ham T, McCulloch W, Dalton M, Reed GA, Baltezor MJ, Jensen RA, Taylor JA, Anant S (2021). Fosciclopirox suppresses growth of high-grade urothelial cancer by targeting the γ-secretase complex. Cell Death Dis.

[112] Dandawate P, Subramaniam D, Panovich P, Standing D, Krishnamachary B, Kaushik G, Thomas SM, Dhar A, Weir SJ, Jensen RA, Anant S (2020). Cucurbitacin B and I inhibits colon cancer growth by targeting the Notch signaling pathway. Sci Rep.

[113] Gounder MM, Rosenbaum E, Wu N, Dickson MA, Sheikh TN, D'Angelo SP, Chi P, Keohan ML, Erinjeri JP, Antonescu CR, Agaram N, Hameed MR, Martindale M, Lefkowitz RA, Crago AM, Singer S, Tap WD, Takebe N, Qin LX, Schwartz GK (2022). A Phase Ib/II Randomized Study of RO4929097, a Gamma-Secretase or Notch Inhibitor with or without Vismodegib, a Hedgehog Inhibitor, in Advanced Sarcoma. Clin Cancer Res.

[114] Sosa Iglesias V, Theys J, Groot AJ, Barbeau LMO, Lemmens A, Yaromina A, Losen M, Houben R, Dubois L, Vooijs M (2018). Synergistic Effects of NOTCH/γ-Secretase Inhibition and Standard of Care Treatment Modalities in Non-small Cell Lung Cancer Cells. Front Oncol.

[115] Jiang C, Cano-Vega MA, Yue F, Kuang L, Narayanan N, Uzunalli G, Merkel MP, Kuang S, Deng M (2017). Dibenzazepine-Loaded Nanoparticles Induce Local Browning of White Adipose Tissue to Counteract Obesity. Mol Ther..

[116] Lobov AA, Boyarskaya NV, Kachanova OS, Gromova ES, Shishkova AA, Zainullina BR, Pishchugin AS, Filippov AA, Uspensky VE, Malashicheva AB (2022). Crenigacestat (LY3039478) inhibits osteogenic differentiation of human valve interstitial cells from patients with aortic valve calcification in vitro. Front Cardiovasc Med.

[117] Borthakur G, Martinelli G, Raffoux E, Chevallier P, Chromik J, Lithio A, Smith CL, Yuen E, Oakley GJ, Benhadji KA, DeAngelo DJ (2021). Phase 1 study to evaluate Crenigacestat (LY3039478) in combination with dexamethasone in patients with T-cell acute lymphoblastic leukemia and lymphoma. Cancer.

